# An unusual case of hypertrophic cardiomyopathy causing right ventricular outflow tract obstruction

**DOI:** 10.1186/1532-429X-16-S1-T9

**Published:** 2014-01-16

**Authors:** Michelle Walkden, Alison M Fletcher, Stephen Harden, James Shambrook, Charles Peebles

**Affiliations:** 1 Cardiothoracic Radiology, University Hospital Southampton, Southampton, Hampshire, UK

## Background

A 14 year old girl was referred for CMRI following an echo demonstrating Left Ventricular hypertrophy and pulmonary stenosis. She presented to Cardiology with atypical chest pain at 13 years and had an exercise tolerance test that induced chest pain. She had a history of a murmur at 6 weeks of age, which was followed up with an echo at 6 months which showed mild pulmonary stenosis.

## Methods

Steady-state free precession (SSFP) imaging was performed to assess ventricular function, along with tagging. Black blood T1 and T2 weighted fast spin echo sequences were acquired for tissue characterisation and phase contrast imaging was used to quantify blood flow. First pass perfusion imaging was performed during the administration of gadolinium and finally Late Gadolinium Enhancement (LGE) imaging was acquired.

## Results

SSFP imaging showed a marked asymmetrical hypertrophy of the crest of the intraventricular septum which was eccentric in nature and protruded extensively into the right ventricle where it caused to a degree of subinfundibular and infundibular narrowing. The Pulmonary Valve appeared a little thickened and domed and without functionally significant stenosis. There was no evidence of systolic anterior motion (SAM) or LVOT obstruction. Black blood imaging did not show any obvious oedema or signal change in the area of hypertrophy. First pass perfusion indicated the area of hypertrophy enhanced relatively normally without any microvascular dysfunction. LGE showed minor patchy scar within the area of maximal hypertrophy and there was no myocardial scar elsewhere. The patient is being treated medically with monitoring for rhythm disturbances and further imaging will be performed at a later date

## Conclusions

We report an unusual case of severe mass like asymmetric hypertrophy of the septum consistent with Hypertrophic Cardiomyopathy (HCM). Right Ventricle involvement in HCM is uncommon and has been described previously by Matsunga et al in 1985, Basarge et al in 1996, Kerekes et al in 2003 and Gullumser et al in 2013.

## Funding

None.

**Figure 1:**
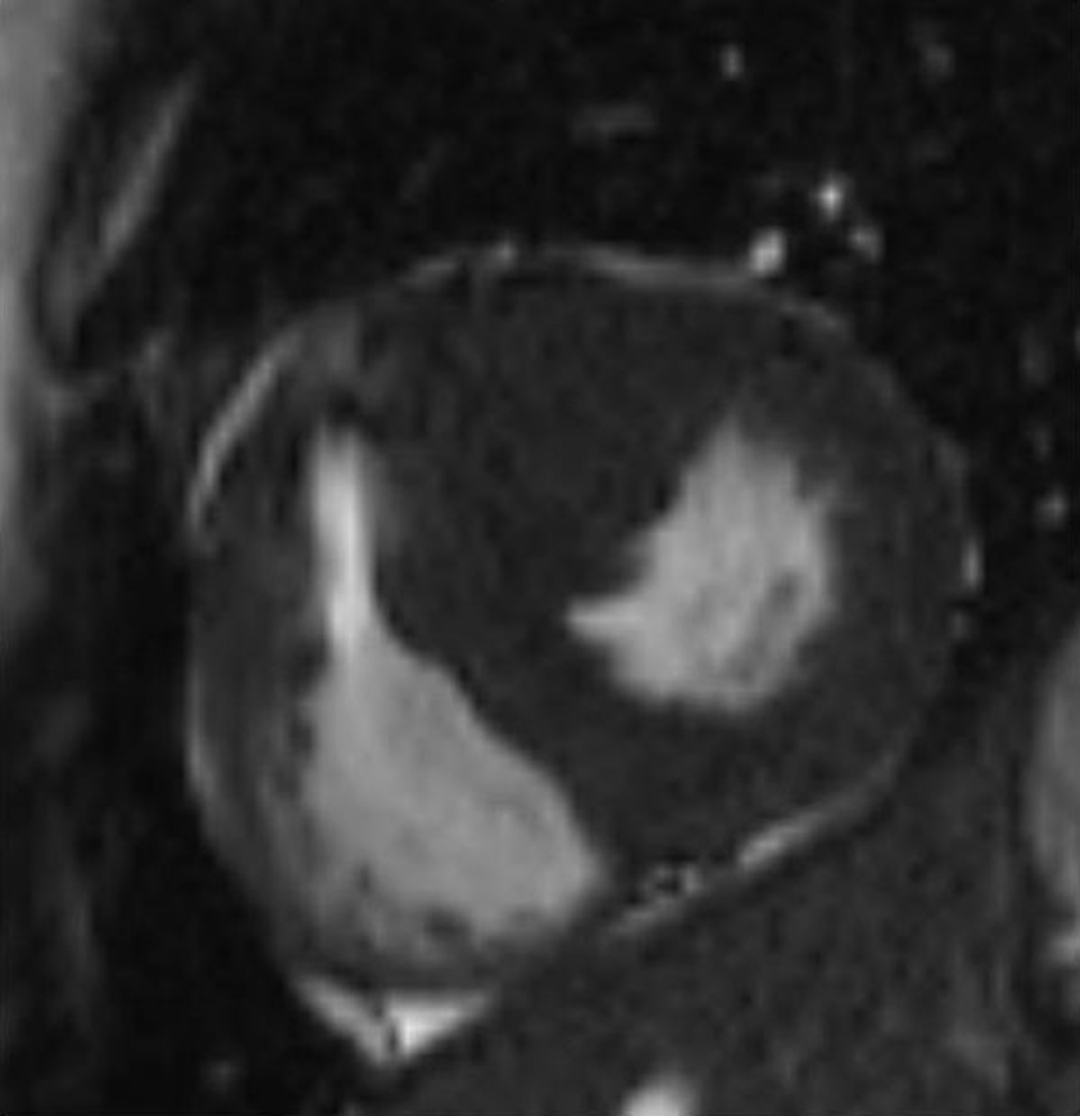
**SSFP to show area of hypertrophy**.

**Figure 2:**
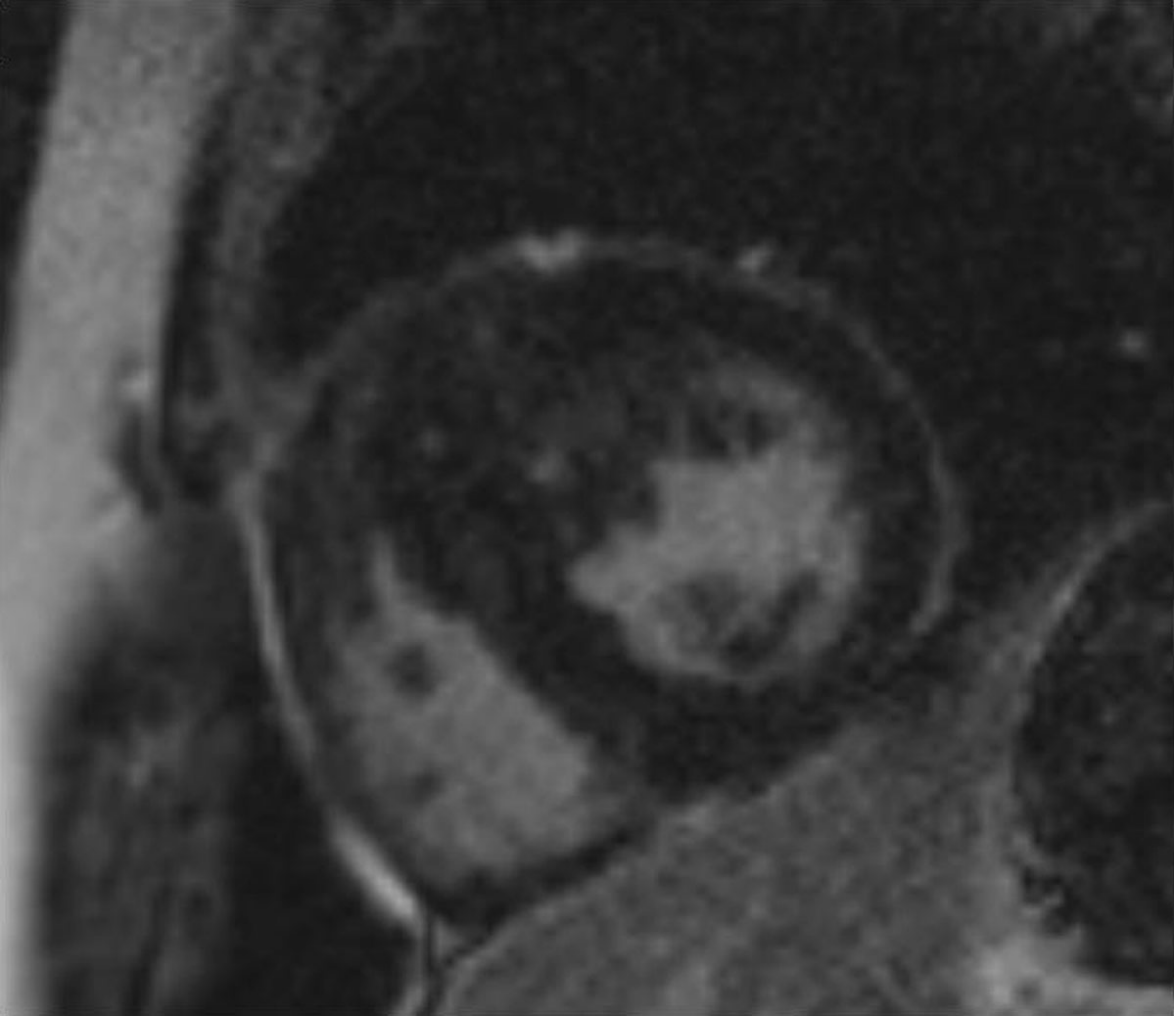
**Late Gadolinium Enhancement showing patchy scar**.

